# REDUCED ENDOTHELIAL FUNCTION IS ASSOCIATED WITH DEEP WHITE MATTER LESION VOLUMES AMONG HEALTHY OLDER ADULTS

**DOI:** 10.1093/geroni/igz038.160

**Published:** 2019-11-08

**Authors:** Regina S Wright, Desiree Bygrave

**Affiliations:** University of Delaware, Newark, Delaware, United States

## Abstract

Reduced endothelial function (EF) is a subclinical cardiovascular disease (CVD) risk factor and precursor to hypertension and atherosclerosis. Among older adults with CVD, reduced EF has been associated with poorer outcomes in a number of cognitive domains, partly explained by the presence of white matter lesion volumes (WMLV) detectable on brain magnetic resonance imaging (MRI). The role of EF as a key, early predictor of brain decrements among older adults without CVD, however, is not well understood. Therefore, the objective of the study was to examine associations between endothelial function and WMLV among cognitively intact older adults free of CVD. A diverse sample of 138 community-based older adults (30.4% male; mean age=68.54y) enrolled in the Healthy Heart & Mind Study underwent cognitive and psychosocial assessment, vascular testing, and brain MRI. Multiple regressions were run to examine associations between endothelial function, as measured by % change in brachial artery flow-mediated dilation (FMD), and MRI-assessed WMLV in brain regions of interest, after controlling for age, sex, race, education, depression, mean arterial pressure, total cholesterol, and hypertension medication use. Results showed a significant inverse association between % FMD change and deep WMLV (p<.05), but no other regions of interest. Results suggest that reduced EF is associated with greater deep WMLV, an outcome variable attributable to small vessel disease and linked to Alzheimer’s disease in previous studies. The implications of this finding for predicting risk for cognitive impairment among healthy older adults will be discussed.

